# Pharmacological Potential of *Arthrospira platensis* in Mitigating Sub-Chronic Colitis: Redox Homeostasis and Gut Microbiota Modulation

**DOI:** 10.3390/cimb47090778

**Published:** 2025-09-19

**Authors:** Meriem Aziez, Betitera Yanat, Cristina Rodriguez-Diaz, Ramona Suharoschi, Romana Vulturar, Simona-Codruta Heghes, Nawel Guenaoui, Awadh M. Ali, Eduardo Garcia-Fuentes, Noureddine Bribi

**Affiliations:** 1 Laboratory of Plant Biotechnology and Ethnobotany, Faculty of Nature and Life Sciences, University of Bejaia, Bejaia 06000, Algeria; meriem.aziez@univ-bejaia.dz (M.A.); betitera.yanat@u-pec.fr (B.Y.); 2 Instituto de Investigación Biomédica de Málaga y Plataforma en Nanomedicina-IBIMA Plataforma BIONAND, 29010 Málaga, Spain; cris.rdrz@gmail.com (C.R.-D.); edugf1@gmail.com (E.G.-F.); 3 Servicio de Aparato Digestivo, Hospital Universitario Virgen de la Victoria, 29010 Málaga, Spain; 4 Molecular Nutrition and Proteomics Lab, Bld. Life Science Institute, Department of Food Science, University of Agricultural Science and Veterinary Medicine, 3-5 Calea Mănăstur, 400372 Cluj-Napoca, Romania; 5 Department of Cell and Molecular Biology, “Iuliu Hațieganu” University of Medicine and Pharmacy, 6 Louis Pasteur St., 400349 Cluj-Napoca, Romania; romanavulturar@gmail.com; 6 Department of Drug Analysis, “Iuliu Hațieganu” University of Medicine and Pharmacy, 6 Louis Pasteur Street, 400349 Cluj-Napoca, Romania; cmaier@umfcluj.ro; 7 Applied Biochemistry Laboratory, Faculty of Natural and Life Sciences, University of Bejaia, Bejaia 06000, Algeria; nawel.guenaoui@univ-bejaia.dz; 8 Department of Pharmaceutical Chemistry, College of Pharmacy, King Saud University, P.O. Box. 2457, Riyadh 11451, Saudi Arabia; aali1@ksu.edu.sa; 9 Centro de Investigación Biomédica en Rehd de Enfermedades Hepáticas y Digestivas (CIBERehd), 29010 Málaga, Spain

**Keywords:** *Arthrospira platensis* (*Spirulina*), DNBS-induced colitis, gut microbiota, microbiota dysbiosis, redox balance, sub-chronic colitis model, 16S rRNA sequencing, complementary therapy, myeloperoxidase (MPO)

## Abstract

Inflammatory bowel diseases (IBDs) are complex disorders involving interconnected immune, oxidative, and microbial dysregulations. *Arthrospira platensis* (Spirulina) is a rich source of bioactive compounds with antioxidant, anti-inflammatory, and immunomodulatory properties. This study investigates the pharmacological efficacy of its aqueous extract (APA) in mitigating 2,4-Dinitrobenzene Sulfonic Acid (DNBS)-induced sub-chronic colitis with a focus on restoring redox balance and modulating gut microbiota composition. APA’s antioxidant capacity was assessed in vitro by 2,2-diphenyl-1-picrylhydrazyl (DPPH), 2,2′-azino-bis (3-ethylbenzthiazoline-6-sulphonic) acid (ABTS) radical scavenging, and metal chelation assays. In vivo, BALB/c mice received two DNBS inductions to establish sub-chronic colitis and were treated with APA (50, 100, and 200 mg/kg). Therapeutic efficacy was assessed through clinical scoring, histopathological assessment, biochemical analysis, and gut microbiota profiling based on 16S rRNA gene sequencing. APA exhibited strong antioxidant activity and significantly attenuated colitis severity, as evidenced by reduced Disease Activity Index (DAI) scores, decreased colon inflammation, suppression of Myeloperoxidase (MPO)-mediated neutrophil infiltration, and modulation of redox biomarkers. Moreover, metagenomic profiling revealed APA-induced modulation of the gut microbiota, mainly through a decreased abundance of pathogenic genera such as *Staphylococcus* and *Enterobacteriaceae*. APA demonstrates potent antioxidant, anti-inflammatory, and microbiota-modulating activities, supporting its potential as a complementary therapy for IBDs and encouraging further clinical studies.

## 1. Introduction

Inflammatory bowel diseases (IBDs) represent a group of chronic, relapsing disorders affecting the gastrointestinal tract, with Crohn’s disease (CD) and ulcerative colitis (UC) as their principal forms [1]. Although their exact etiology remains unclear, IBDs are recognized as multifactorial disorders resulting from complex interactions between genetic predisposition, environmental stimuli, immune system dysregulation, and intestinal microbiota alterations [2,3]. These idiopathic inflammatory conditions of the gastrointestinal tract have distinct pathological and clinical characteristics, reflecting their complex underlying mechanisms [4]. Despite the availability of therapeutic options such as corticosteroids, immunosuppressants, and anti-inflammatory agents, current treatments primarily aim to alleviate symptoms and suppress inflammation [5,6,7]. However, they do not effectively address the underlying pathogenic mechanisms and are frequently associated with adverse effects and variable therapeutic responses, limiting their long-term use [8]. Therefore, there is an urgent need to develop new therapeutic strategies that are both effective and better tolerated [9,10].

Given the limitations of current pharmacological treatments, there is a growing interest in exploring alternative therapeutic strategies for IBD. In this context, experimental models of colitis have become essential tools for elucidating the complex pathophysiology of IBD and for testing novel therapeutic approaches [11,12]. Among these, natural compounds, particularly those derived from cyanobacteria, have attracted considerable attention due to their well-established antioxidant and anti-inflammatory activities. These bioactive molecules are emerging as promising nutraceuticals capable of modulating key pathways involved in IBD pathogenesis, offering potential alternatives or adjuncts to conventional pharmacological therapies [13,14,15].

*Arthrospira platensis*, commonly known as Spirulina, is a cyanobacterium with a long-standing history of use as a dietary supplement and a source of natural bioactive compounds [16,17]. This filamentous organism, belonging to the Oscillatoriaceae family, has garnered considerable scientific interest due to its complex molecular profile, including phycobiliproteins, essential fatty acids, polysaccharides, and phenolic compounds, which underpin its wide-ranging biological activities [18,19]. Ease of cultivation under controlled conditions, along with a high content of bioactive metabolites with recognized therapeutic relevance, *A. platensis* exhibits a wide range of biological activities, including antioxidant, anti-inflammatory, and antimicrobial effects, which contribute to its health-promoting potential [20,21,22,23]. These attributes make it a promising adjuvant therapy for managing various disorders, particularly chronic diseases such as IBD.

To the best of the authors’ knowledge, studies evaluating the aqueous extract of *A. platensis* have been limited to early-stage colitis models, with only a single investigation exploring its impact on gut microbiota composition in an acute Dextran Sulfate Sodium (DSS)-induced colitis model [24]. However, no research has yet addressed its potential in more advanced and complex models of colitis, such as 2,4-Dinitrobenzene Sulfonic Acid (DNBS)-induced sub-chronic colitis, and its ability to modulate both redox homeostasis and gut microbiota simultaneously.

Therefore, this study aimed to investigate the therapeutic potential of *A. platensis* aqueous (APA) extract by integrating in vitro antioxidant assays and an in vivo murine model of chronic colitis induced by DNBS. The evaluation encompassed clinical scoring, macroscopic and histopathological assessment of colonic tissues, quantification of pro-inflammatory and redox biomarkers, as well as 16S rRNA-based profiling of the gut microbiota. This integrative approach provides an in-depth evaluation of the effects of APA extracts, underscoring their potential as a comprehensive therapeutic option for managing IBD.

## 2. Materials and Methods

### 2.1. Biomass and Reagents

Dried biomass of *A. platensis* was obtained from the Algae Culture Laboratory, Kasdi Merbah University, Ouargla, Algeria. All chemical reagents used in this study were purchased from Sigma-Aldrich (Madrid, Spain). The DNeasy Blood and Tissue Kit was obtained from QIAGEN (Hilden, Germany) for DNA extraction procedures.

### 2.2. Preparation of A. platensis Aqueous Extract

The APA extract was prepared according to a previously described method with slight modifications [25]. Briefly, 10 g of powdered *A. platensis* biomass was macerated in distilled water at a 1:10 (*w*/*v*) ratio under continuous agitation at room temperature for 24 h. The resulting mixture was centrifuged (Sigma 3-16 L, 172577, Darmstadt, Germany) at 3500 rpm for 10 min at 4 °C to remove insoluble materials. The supernatant was then filtered through Whatman No. 1 filter paper and dried at a temperature not exceeding 40 °C.

### 2.3. Free Radical Scavenging Activities

The antiradical potential of the APA extract was evaluated using 2,2-diphenyl-1-picrylhydrazyl (DPPH)^•^ and 2,2′-azino-bis (3-ethylbenzthiazoline-6-sulphonic acid) (ABTS)^•+^ assays, following the protocols described by Chaves et al. [26] and Bibi Sadeer et al. [27], respectively. For both methods, various concentrations of the extract (0.1–1 mg/mL) were incubated with the respective radicals, and the reduction in absorbance was measured at 517 nm (DPPH^•^) and 734 nm (ABTS^•+^). Antioxidant capacity was expressed as IC_50_, indicating the concentration of the extract required to scavenge 50% of the radicals. Trolox and ascorbic acid served as positive controls.

### 2.4. Ferrous Ion Chelating Activity

The metal-chelating activity of the APA extract was assessed according to the method of Chai et al. [28], based on the inhibition of the Fe^2+^–ferrozine complex formation. The absorbance of the reaction mixture was recorded at 562 nm, and the chelation capacity was expressed as IC_50_, corresponding to the concentration of extract required to chelate 50% of ferrous ions. Trolox and ascorbic acid were used as reference compounds.

### 2.5. Animals and Grouping

The experimental protocol was carried out in accordance with Directive 2010/63/EU of 22 September 2010 and was approved by the local Ethics Committee of the Laboratory of LBVE, University of Bejaia (Ref. No. CE-LBVE-2024-112). A total of thirty-five female BALB/c mice, weighing 20–25 g, obtained from the Pasteur Institute (Algiers, Algeria), were housed in standard cages under an equal light/dark cycle and were given free access to water and food. Animals were divided randomly into five groups as follows. Group I: a non-colitic group and four DNBS-induced colitic groups, including group II: an untreated colitic group; groups III, IV, and V were treated orally for seven days with three doses of APA extract (50, 100, and 200 mg/kg), respectively.

### 2.6. Induction and Assessment of Colitis in Mice

**Figure 1:**
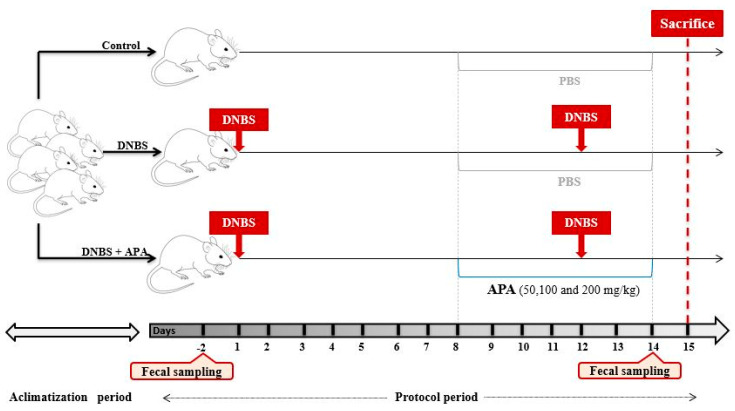
Experimental design of DNBS-induced sub-chronic colitis in BALB/c mice and treatment with multiple doses of *A. platensis* aqueous extract (APA).

The protocol for DNBS-induced colitis, as detailed in Figure 1, follows the method previously reported by Martín et al. [29]. Briefly, colitis was induced through intrarectal injection, administering 2 doses of DNBS with a 12-day interval between each dose. The Disease Activity Index (DAI) was determined by monitoring daily variations in body weight and clinical manifestations of the disease, including bleeding stools, stool consistency, wet anus, piloerection, and hypoactivity, beginning from the second induction. Fecal samples were collected once from the healthy control group and twice from all DNBS-treated groups, before induction and after colitis was established. Following a 14-day period from the initial DNBS induction, the animals were euthanized and dissected. The entire colon was excised, weighed, and its length was measured.

### 2.7. Histological Examination of the Colon

Colon specimens were fixed in 4% buffered formaldehyde for histological examination. After fixation, cross-sections were selected and embedded in paraffin. Subsequently, 5 µm full-thickness sections were obtained at various levels, mounted on silane-coated glass slides, and stained with hematoxylin–eosin for histological evaluation. The sections were then observed using a Leica microscope (Leica DM1000, Wetzlar, Germany) for analysis.

### 2.8. Evaluation of Polynuclear Neutrophil Infiltration

Colon tissues were first homogenized and sonicated, and the post-mitochondrial supernatant (PMS) was collected according to the protocol of Merakeb et al. [30]. Polymorphonuclear neutrophil infiltration was then assessed by measuring Myeloperoxidase (MPO) activity, as described by Aziez et al. [31]. Briefly, 0.1 mL of PMS was combined with 2.9 mL of 50 mM phosphate buffer (pH 6.0), containing 0.167 mg/mL O-dianisidine hydrochloride and 0.0005% hydrogen peroxide. The change in absorbance was monitored at 460 nm over a 3 min period. MPO activity was expressed as mM per minute per gram of colon tissue.

### 2.9. Redox Biomarkers Analysis

#### 2.9.1. Nitric Oxide (NO) Levels

Nitrite concentrations, as indicators of NO production, were quantified using the Griess assay [32]. PMS samples were deproteinized with 10% TCA, reacted with Griess reagent, and absorbance was read at 543 nm. Results were expressed as µM/100 mg tissue using a sodium nitrite (NaNO_2_) standard curve (ranging from 1 to 128 µM).

#### 2.9.2. Malondialdehyde (MDA) Levels

Lipid peroxidation was quantified by measuring MDA levels following the thiobarbituric acid reactive substances (TBARS) assay [33]. PMS samples were mixed with TCA (35%) and incubated at 4 °C for 1 h, followed by centrifugation at 1466× *g* for 10 min. The supernatant was reacted with TBA (0.8%) under acidic conditions, heated at 95 °C for 1 h, and then cooled to 4 °C. The absorbance was measured at 532 nm. MDA levels were calculated using an extinction coefficient of 1.56 × 10^5^ M^−1^·cm^−1^ and expressed as µM/g of colon tissue.

#### 2.9.3. Catalase (CAT) Activity

CAT activity was assessed in supernatants of each sample using the protocol described by Avula et al. [34]. The enzymatic reaction was initiated by adding 1.95 mL of 0.2% H_2_O_2_ to 50 μL of PMS diluted in phosphate buffer (50 mM, pH 7). The degradation of hydrogen peroxide was measured kinetically at 240 nm for 3 min. The results were expressed in mM per minute per g of colon tissue.

#### 2.9.4. Reduced Glutathione (GSH) Levels

The determination of GSH content in the PMS of tissue homogenates was performed according to the method of Rathore et al. [35]. The reduction of 5,5′-dithiobis 2-nitrobenzoic acid (DTNB) by glutathione produces 2-nitro-5-thiobenzoic acid (TNB), which absorbs at 405 nm. The results were expressed in µM per 100 mg of protein. Bovine serum albumin (BSA) was used as a reference standard for making a calibration curve concentration (ranging from 0 to 100 µg.mL^−1^) [36].

### 2.10. Metagenomic Analysis of the Fecal Microbiota

Total genomic DNA was extracted from the stool samples using the QIAGEN Science (Hilden, Germany), following the manufacturer’s recommendations. DNA was eluted in DNase/RNase-free water, and its concentration and purity were evaluated using a NanoDrop ND-1000 spectrophotometer (NanoDrop Technologies, Inc., Wilmington, DE, USA). Next, libraries were prepared by amplifying the V1–V3 regions of the 16S rRNA bacterial gene, and sequencing bioinformatics, ordination and statistical analysis were performed as previously described [37]. Briefly, amplification of the V1-V3 regions of the 16S rRNA bacterial gene was performed using the primers 5′-GAGAGTTTGATYMTGGCTCAG-3′ (forward) and 5′-ACCGCGGCTGCTGGCAC-3′ (reverse) with overhang adapters, allowing amplification of diverse bacterial sequences. Amplicons were purified using the Agencourt AMPure XP bead kit (Beckman Coulter, Pasadena, CA, USA) and indexed with Nextera XT index primers 1 and 2 (Illumina, San Diego, CA, USA). Amplicon concentrations were quantified using Quant-IT PicoGreen (Thermo Fisher Scientific, Waltham, MA, USA) and diluted to 10 ng/μL. DNA samples were further quantified by qPCR using the KAPA SYBR^®^ FAST qPCR Kit (Kapa Biosystems, Wilmington, MA, USA). Samples were normalized, pooled, and sequenced on the Illumina MiSeq platform (v3 reagents, paired-end reads) by GIGA Genomics (Liège, Belgium). Positive controls consisted of a defined bacterial community with known proportions of *Carnobacterium maltaromaticum*, *Lactococcus lactis* subsp. *cremoris*, *Leuconostoc carnosum*, *Pseudomonas aeruginosa*, and *Streptococcus thermophilus*. Negative controls were used in their entirety for DNA extraction, library preparation, and sequencing.

### 2.11. Statistical Analysis

Data are presented as mean ± standard deviation (SD). Statistical analyses were performed using GraphPad Prism 8.0.2.263 (GraphPad Software, San Diego, CA, USA). Group comparisons were conducted using one-way ANOVA, followed by Dunnett’s test for in vivo data and Tukey’s post hoc test for in vitro assays. For metagenomic data, Good’s coverage and the Chao1 richness estimator were calculated using Mothur v1.44.3. Two-group comparisons were analyzed using the Wilcoxon matched-pairs signed rank test, while comparisons among three or more groups were assessed with the Kruskal–Wallis test followed by Benjamini–Hochberg false discovery rate (FDR) correction. A *p*-value < 0.05 was considered statistically significant.

## 3. Results

The High-Performance Liquid Chromatography coupled with Diode Array Detection and Electrospray Ionization Mass Spectrometry (HPLC-DAD-ESI-MS) analytical method used for the characterization of phenolic compounds in the APA extract was previously described in our earlier study [31]. The chromatogram and detailed composition of the extract are provided in Figure S1 and Table S1. Phenolic acids were identified as the predominant class of bioactive compounds, with a total content of 8.859 mg/g extract. The phenolic profile was mainly composed of hydroxybenzoic acids and related derivatives, including pyrogallol, gallic acid, and other benzoic acid isomers.

### 3.1. In Vitro Antioxidant Capacity

**Figure 2:**
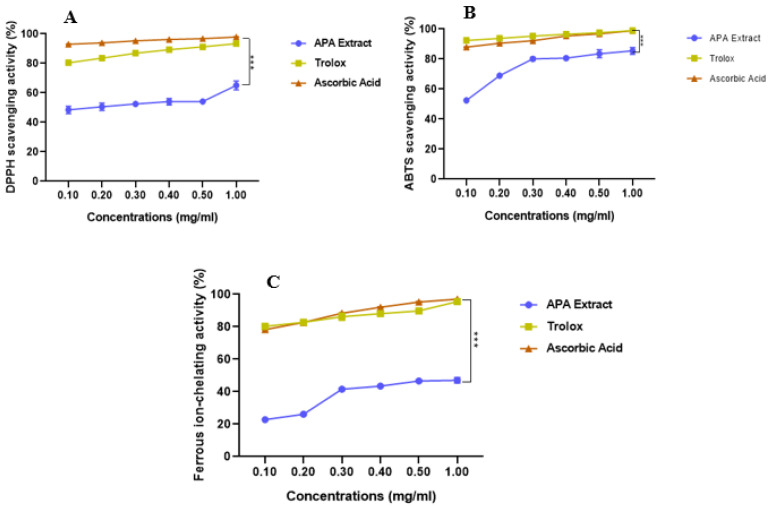
Antioxidant capacity of *A. platensis* aqueous extract (APA) in comparison with standards. (**A**) DPPH**•** radical scavenging activity; (**B**) ABTS**•**^+^ radical scavenging activity; (**C**) ferrous ion chelating activity. Data represent mean ± standard deviation (SD) from three independent experiments (*n* = 3). Statistical significance was assessed by one-way ANOVA followed by Tukey’s post hoc test. *** *p* < 0.001 vs. the standards.

As shown in Figure 2A–C and Table 1, APA exhibited vigorous antioxidant activity in a dose-dependent manner. At 1 mg/mL, it scavenged DPPH• and ABTS•^+^ radicals by 65.04 ± 2.98% and 85.44 ± 2.27%, respectively. The IC_50_ values were 0.166 ± 0.00 mg/mL for DPPH• and 0.118 ± 0.00 mg/mL for ABTS•^+^, compared with Trolox (IC_50_: 0.081 ± 0.00 mg/mL for DPPH• and 0.068 ± 0.00 mg/mL for ABTS•^+^) and ascorbic acid (IC_50_: 0.067 ± 0.00 mg/mL for DPPH• and 0.072 ± 0.00 mg/mL for ABTS•^+^). Although these differences were statistically significant, APA exhibited a potent antioxidant capacity.

Additionally, APA displayed notable ferrous ion chelation, reaching 48.66 ± 0.43% at 1 mg/mL with an IC_50_ of 0.939 ± 0.05 mg/mL, which is significantly lower than that of Trolox (0.081 ± 0.00 mg/mL) and ascorbic acid (0.083 ± 0.00 mg/mL). This chelating ability is crucial in limiting the formation of ROS through metal-catalyzed redox reactions.

### 3.2. Effects of APA Extract on the DAI and Colon Morphology

**Figure 3:**
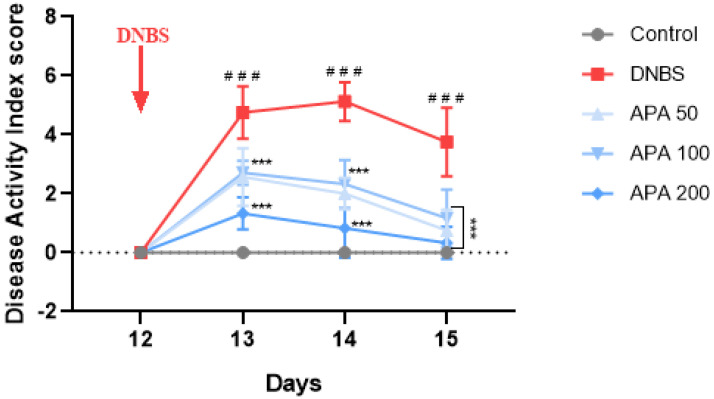
Impact of oral administration of *A. platensis* aqueous extract (APA) on the Disease Activity Index (DAI) in a DNBS-induced sub-chronic colitis model in BALB/c mice. Mice received APA extract at doses of 50, 100, and 200 mg/kg. Data are presented as mean ± standard deviation (SD) (*n* = 7 per group). Statistical analysis was conducted using one-way ANOVA followed by Dunnett’s multiple comparisons test. *** *p* < 0.001 vs. the DNBS group; ### *p* < 0.001 vs. the control group.

To evaluate the anti-inflammatory potential of APA extract in sub-chronic DNBS-induced colitis, both the DAI and colon weight/length ratio were assessed. The DAI was significantly increased in the untreated colitic group compared with the non-colitic group, indicating marked disease progression. However, oral administration of APA extract at 50, 100, and 200 mg/kg significantly attenuated this increase (Figure 3).

**Figure 4:**
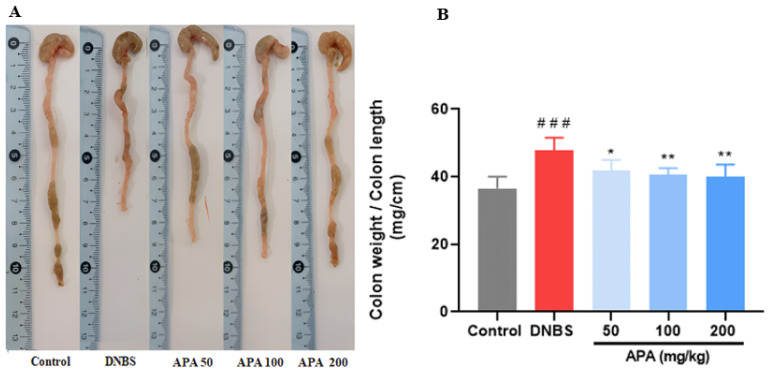
Effect of oral administration of *A. platensis* aqueous extract (APA) on colon morphology and weight/length ratio in DNBS-induced sub-chronic colitis in BALB/c mice. (**A**) Representative macroscopic images of the colon; (**B**) colon weight-to-length ratio. Mice received APA extract at doses of 50, 100, and 200 mg/kg. Data are presented as mean ± standard deviation (SD) (*n* = 7 per group). Statistical analysis was performed using one-way ANOVA followed by Dunnett’s multiple comparisons test. * *p* < 0.05, ** *p* < 0.01 vs. the DNBS group; ### *p* < 0.001 vs. the control group.

Moreover, the colon weight-to-length ratio, a macroscopic indicator of inflammation, was significantly higher in the untreated colitic group (47.85 ± 3.70) compared with the healthy control group (36.55 ± 3.44; *** *p* < 0.001). Treatment with APA extract resulted in a significant reduction in this parameter, with values of 41.98 ± 2.95, 40.66 ± 1.89, and 39.98 ± 3.66 at doses of 50, 100, and 200 mg/kg, respectively (Figure 4A,B), reflecting an improvement in colonic inflammation.

### 3.3. Effect of APA on Colonic Lesions

**Figure 5:**
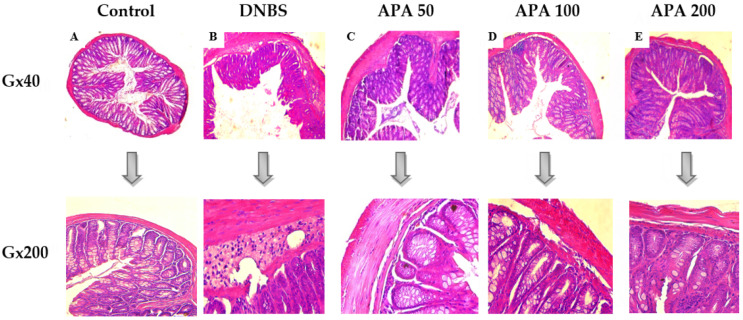
Histological evaluation of colonic tissue following *A. platensis* aqueous extract (APA) treatment in DNBS-induced sub-chronic colitis in mice. Representative photomicrographs of hematoxylin–eosin-stained transverse colon sections, analyzed under light microscopy (G×40 and G×200), show (**A**) normal architecture in the control group, (**B**) extensive histopathological alterations in the untreated colitic group, and (**C**–**E**) dose-dependent histological improvements in groups treated with APA extract (50, 100, and 200 mg/kg, respectively). DNBS: 2,4-Dinitrobenzene Sulfonic Acid.

Histopathological assessment revealed the protective efficacy of the APA extract against DNBS-induced colonic injury. In the non-colitic control group, no histological alterations were observed, indicating healthy colon tissue (Figure 5A). In contrast, the untreated colitic group exhibited severe structural damage, which was characterized by pronounced submucosal edema, extensive infiltration of inflammatory cells in the submucosa, and disruption of crypt integrity accompanied by the presence of granulomas, effectively mimicking the pathological features of Crohn’s disease (Figure 5B) [1]. Treatment with APA extract at doses of 50, 100, and 200 mg/kg led to marked improvements in the histological appearance of the mucosa, submucosa, and muscularis mucosa. This treatment preserved the structural integrity of the crypts, reduced submucosal edema, and mitigated the infiltration of inflammatory cells without causing any architectural damage (Figure 5C–E). These findings indicate that APA extract exerts a strong anti-inflammatory effect, mitigating DNBS-induced histopathological alterations and preserving colonic structural integrity.

### 3.4. Effect of APA Extract on Polymorphonuclear Cells Infiltration

**Figure 6:**
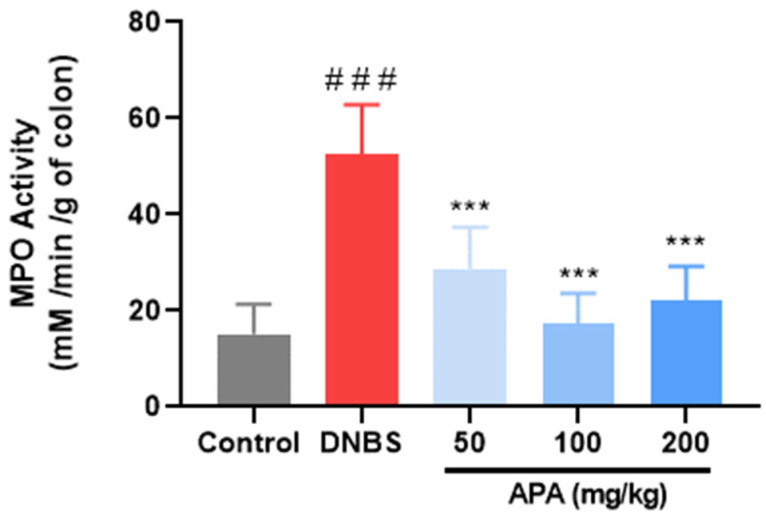
Effect of *A. platensis* aqueous extract (APA) on myeloperoxidase (MPO) activity in DNBS-induced sub-chronic colitis in BALB/c mice. Mice were treated orally with APA extract at doses of 50, 100, and 200 mg/kg. Data are presented as mean ± standard deviation (SD) (*n* = 7 per group). Statistical analysis was performed using one-way ANOVA followed by Dunnett’s multiple comparisons test. *** *p* < 0.001 vs. the DNBS group; ### *p* < 0.001 vs. the control group.

MPO activity, a well-established biochemical marker of neutrophil accumulation within inflamed tissues, was assessed to evaluate the extent of polymorphonuclear cell infiltration [38]. The MPO levels were significantly increased in the untreated colitic group (52.53 ± 9.39 mM/Min/g of colon) compared with the non-colitic control (11.70 ± 3.69 mM/Min/g of colon; *** *p* < 0.001), reflecting intense neutrophil-mediated inflammation (Figure 6). Oral administration of APA extract at doses of 50, 100, and 200 mg/kg led to a significant reduction in MPO activity, with values of 28.75 ± 7.78, 17.35 ± 5.42, and 22.12 ± 6.14 mM/Min/g of colon, respectively. These findings highlight the capacity of APA extract to attenuate neutrophilic infiltration and thereby mitigate colonic inflammatory responses.

### 3.5. Effects of APA Extract on Redox Biomarkers

**Figure 7:**
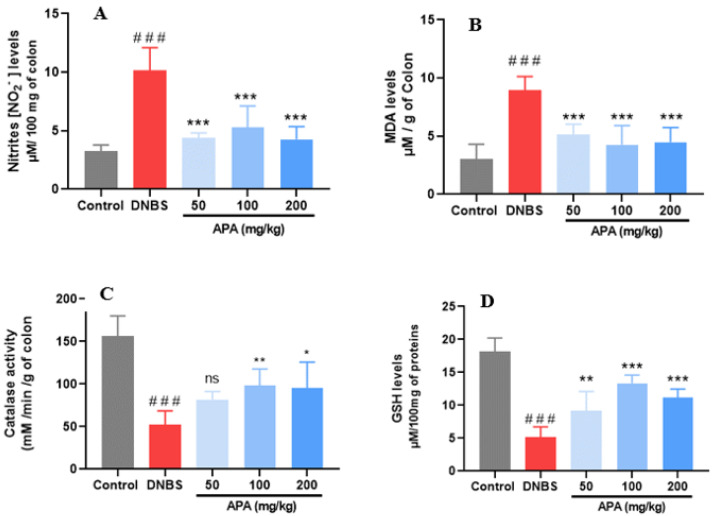
Effects of *A. platensis* Aqueous Extract (APA) on redox biomarkers in DNBS-induced sub-chronic colitis in BALB/c mice. (**A**) Nitrite (NO_2_^−^) levels; (**B**) malondialdehyde (MDA) content; (**C**) catalase (CAT) activity; and (**D**) reduced glutathione (GSH) levels. Mice were treated orally with APA extract at doses of 50, 100, and 200 mg/kg. Data are presented as mean ± standard deviation (SD) (*n* = 7 per group). Statistical analysis was performed using one-way ANOVA followed by Dunnett’s multiple comparisons test. ns, *p* > 0.05, * *p* < 0.05, ** *p* < 0.01, *** *p* < 0.001 vs. the DNBS group; ### *p* < 0.001 vs. the control group.

Biochemical assessment of colonic tissues was conducted to investigate the modulatory effects of APA extract on redox biomarkers associated with DNBS-induced sub-chronic colitis. NO level, assessed indirectly by measuring its stable end-product (nitrite), a key inflammatory mediator involved in vasodilation, increased vascular permeability, and intestinal tissue injury, was significantly increased in the untreated colitic group (10.14 ± 1.95 µM/100 mg of colon) relative to the non-colitic control group (3.26 ± 0.52 µM/100 mg of colon; *** *p* < 0.001) (Figure 7A). Oral administration of APA extract at 50, 100, and 200 mg/kg significantly reduced NO levels to 4.35 ± 0.45, 4.87 ± 1.66, and 4.25 ± 1.09 µM/100 mg, respectively, indicating attenuation of nitrosative stress. Lipid peroxidation, assessed by malondialdehyde (MDA) quantification, was significantly increased in the untreated colitic group (8.94 ± 1.18 µM/g tissue) compared with the healthy controls (3.04 ± 1.26 µM/g of colon; *** *p* < 0.001), reflecting excessive reactive oxygen species (ROS) generation (Figure 7B). APA extract treatment resulted in a decrease in MDA levels, yielding 5.12 ± 1.23, 4.27 ± 0.08, and 4.49 ± 0.62 µM/g of colon for the respective doses, suggesting effective inhibition of lipid peroxidation.

Furthermore, DNBS-induced colitis caused a significant depletion of endogenous antioxidant defenses, as evidenced by reduced catalase activity (52.00 ± 16.57 mM/min/g of colon) and reduced GSH content (5.19 ± 1.54 µM/100 mg of protein compared with the non-colitic group (CAT: 156.21 ± 23.81; GSH: 18.16 ± 2.06; *** *p*< 0.001) (Figure 7C,D). Treatment with APA extract restored these antioxidant parameters. Catalase activity increased to 81.69 ± 8.78, 98.19 ± 17.97, and 95.52 ± 26.31 mM/min/g of colon, while GSH levels were elevated to 9.18 ± 1.39, 13.35 ± 1.28, and 11.13 ± 2.60 µmol/100 mg of protein for 50, 100, and 200 mg/kg doses, respectively.

### 3.6. Metagenomic Profiling of Colonic Microbiota

Metagenomic analysis of fecal samples was conducted to evaluate the impact of the APA extract on gut microbiota composition before and after DNBS-induced sub-chronic colitis. Microbial richness (Chao1 index), alpha diversity (inverse Simpson index), and evenness (Simpson derived index) did not show statistically significant differences between pre- and post-DNBS-induced sub-chronic colitis across all studied groups.

**Figure 8:**
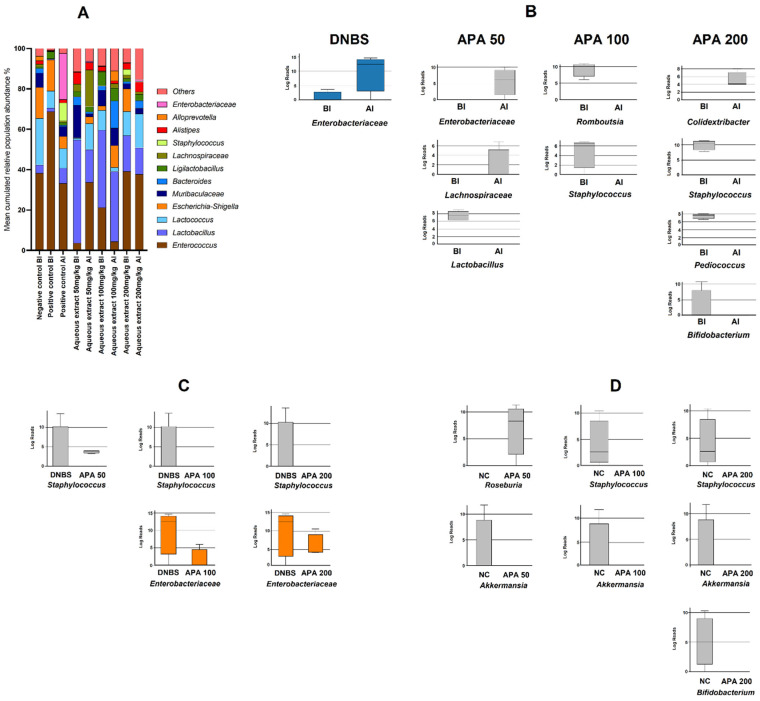
(**A**) Relative abundance of the 12 most prevalent taxa identified among all those with a prevalence greater than 1% in the fecal microbiota of non-colitic mice group (negative control, NC), untreated DNBS-induced colitic group (positive control) and DNBS-induced colitic groups treated with *A. platensis* aqueous extract (APA) at doses of 50, 100 and 200 mg/kg before (BI) and after (AI) inflammation. (**B**) Significant bacterial taxa were identified after pairwise comparison in the before–after treatment comparisons within each of the groups studied. (**C**) Significant bacterial taxa identified in the comparison between the untreated DNBS-induced colitic group and APA-treated groups after treatments. (**D**) Significant bacterial taxa were identified in the comparison between the non-colitic group (negative control) and the APA-treated groups after treatment. Results obtained after DESeq2 with Benjamini–Hochberg FDR corrections. *Enterobacteriaceae* is shown in color since it is the 12th most abundant bacterial group in the total number of samples analyzed. Log Reads: Log2 relative normalized abundance.

Figure 8A illustrates the 12 most prevalent taxa identified among all those with a prevalence greater than 1% in non-colitic, untreated colitic, and APA-treated colitic mice (50, 100, and 200 mg/kg)**.** By comparing samples within each group before and after sub-chronic colitis induction, a significant increase in Enterobacteriaceae was found in the untreated colitic group following sub-chronic DNBS-induced colitis (Figure 7B). Treatment with APA at 50 mg/kg decreased *Lactobacillus_HT002* while increasing *Lachnospiraceae_ASF356* and *Enterobacteriaceae* (Figure 7B). APA at 100 mg/kg decreased *Romboutsia* and *Staphylococcus* (Figure 8B). At 200 mg/kg, APA treatment increased *Colidextribacter* and decreased *Staphylococcus*, *Bifidobacterium*, and *Pediococcus* (Figure 8B).

Following sub-chronic DNBS-induced colitis, APA-treated groups exhibited distinct microbial profiles compared with the untreated colitic group. The abundance of *Staphylococcus* was significantly reduced with all APA doses (50, 100, and 200 mg/kg), while *Enterobacteriaceae* was significantly reduced with APA 100 and 200 mg/kg doses (Figure 8C).

APA extract treatment produced additional changes compared with the non-colitic group. A decline in *Akkermansia* was observed at doses of 50, 100, and 200 mg/kg, while *Staphylococcus* exhibited a decrease only at the 100 and 200 mg/kg doses (Figure 8D). In addition, a significant increase in the abundance of *Roseburia* was observed at a dosage of 50 mg/kg, accompanied by a decline in *Bifidobacterium* at 200 mg/kg (Figure 8D).

## 4. Discussion

This study provides strong evidence for the therapeutic potential of APA extract in mitigating DNBS-induced sub-chronic colitis, through a multitargeted mechanism involving antioxidant, anti-inflammatory, and gut microbiota-modulating activities. Using a well-established murine model that mimics key features of human Crohn’s disease, APA administration alleviated the clinical, histological, biochemical, and microbial disturbances associated with intestinal inflammation.

In vitro analyses revealed that the APA extract exhibits a strong antioxidant capacity, as demonstrated by its ability to scavenge free radicals in both DPPH and ABTS assays, as well as through its metal ion chelation activity. This antioxidant effect is likely related to the high content of bioactive compounds in the extract, notably phycocyanins, phenolic acids, and other phytochemicals known for their redox-modulating properties [39,40]. Such observations are consistent with previous findings by Shalaby and Shanab [41] and Bellahcen et al. [42], who reported that the antioxidant properties of Spirulina are primarily attributed to its unique composition of these molecules. Furthermore, the metal ion chelation ability observed in this study suggests that APA may mitigate oxidative damage by reducing metal-catalyzed ROS generation, an essential pathway in oxidative stress-related tissue injury.

In vivo, our results clearly demonstrate that oral administration of the APA extract at doses of 50, 100, and 200 mg/kg significantly alleviated DNBS-induced colitis in mice, as evidenced by reductions in DAI, improvements in histopathological scores, and restoration of colonic architecture. Additionally, this treatment resulted in a significant reduction in MPO activity, NO, and MDA levels, alongside an increase in antioxidant defenses such as catalase and GSH, indicating a strong anti-inflammatory and antioxidant effect. These protective outcomes underscore the ability of APA extract to mitigate local inflammation, reduce oxidative damage, and promote tissue healing in the colonic mucosa. Such findings align with the observed histological restoration, including decreased mucosal ulceration, reduced neutrophilic infiltration, and partial recovery of crypt structures, further supporting the tissue-protective role of APA extract.

These effects were corroborated by histological observations and biochemical analyses, demonstrating APA’s capacity to counteract the pathological consequences of DNBS-induced inflammation. Our findings are consistent with previous research highlighting the anti-inflammatory potential of *A. platensis*, particularly through the downregulation of pro-inflammatory mediators such as cyclooxygenase-2 (COX-2), prostaglandin E2 (PGE2), inducible nitric oxide synthase (iNOS), tumor necrosis factor-alpha (TNF-α), and interferon-gamma (IFN-γ) in the intestinal environment [43,44,45,46].

Importantly, our study extends these observations by demonstrating that APA extract not only modulates inflammatory pathways but also restores redox balance, an essential aspect often overlooked in IBD research. This evidence supports the potential of A. platensis as a promising therapeutic agent for managing IBD.

Metagenomic analysis revealed essential insights into the effects of APA on gut microbiota composition. Our study demonstrates that oral administration of APA extract exerts a selective modulatory effect on the gut microbiota of untreated colitic mice, particularly by reducing pathogenic taxa and partially restoring beneficial bacteria. Our results showed that DNBS-induced sub-chronic colitis led to a significant expansion of pro-inflammatory taxa such as *Enterobacteriaceae*, in line with previous reports of dysbiosis in Crohn’s disease models. Conversely, APA treatment modulated this dysbiotic profile, notably reducing *Staphylococcus* and *Enterobacteriaceae* in higher doses (100 and 200 mg/kg), while enriching beneficial genera like *Roseburia* at lower doses. The observed antimicrobial effect of APA against *Staphylococcus* is particularly interesting, as it may be attributed to the presence of specific bioactive compounds, such as phycocyanins, phenolic acids, and other metabolites, which are known to interfere with pathogen membrane integrity and functionality [47,48]. This selective antimicrobial activity highlights the potential of APA as a targeted modulator of gut microbiota composition, rather than a broad-spectrum antimicrobial agent that could disrupt commensal populations.

Chronic colitis, particularly in Crohn’s disease, is associated with microbial dysbiosis characterized by reduced species diversity and the expansion of pathogenic taxa [49]. Although our alpha diversity metrics (Chao1, Inverse Simpson) did not show significant changes, the specific alterations in key bacterial taxa suggest a compositional shift rather than a global loss of diversity, a pattern also observed in some human IBD cohorts. This finding suggests that APA extract exerts a corrective effect on microbiota composition by targeting specific dysbiotic signatures rather than indiscriminately altering microbial diversity.

These changes are known to impair intestinal barrier integrity and promote pathological immune responses, particularly in individuals with a genetic predisposition [37,49]. Previous studies have shown that genera such as *Lachnospiraceae*, *Clostridiales*, *Enterobacteriaceae*, *Escherichia-Shigella*, and *Peptostreptococcaceae* tend to increase in Crohn’s disease patients, while beneficial taxa like *Ruminococcaceae*, *Faecalibacterium prausnitzii*, *Bacteroidetes*, and *Bifidobacterium* breve decrease [50]. Our results align with this pattern, showing an increase in pro-inflammatory taxa in colitic mice and a reversal of these trends following APA treatment, particularly at higher doses.

These beneficial microbes exert anti-inflammatory effects through the production of short-chain fatty acids (SCFAs), especially butyrate, and immune modulation, whereas harmful taxa contribute to intestinal inflammation [51,52]. The partial restoration of beneficial taxa such as *Roseburia*, alongside the decline in pro-inflammatory groups like *Staphylococcus*, observed in our APA-treated groups, suggests that APA extract may help rebalance the gut microbiota toward a more anti-inflammatory and health-associated profile.

However, some limitations must be acknowledged. Notably, the short duration of the treatment protocol and the early timing of fecal sampling may have limited the detection of deeper or more stable microbiota shifts. As suggested by Nagata et al. [51], longer treatment periods and delayed sampling, ideally at least 14 days post-treatment, may be necessary to fully capture the impact of APA on gut microbial communities. Furthermore, this study did not investigate specific molecular pathways such as cytokine modulation or epithelial barrier reinforcement, which could elucidate the mechanisms behind the observed improvements. While our microbiota analysis highlighted key genera changes, integrating functional metagenomics or metabolomics in future studies would provide a more comprehensive understanding of host–microbiota interactions and metabolic outputs.

## 5. Conclusions

This study underscores the efficacy of APA in mitigating key features of chronic DNBS-induced colitis by improving clinical outcomes, preserving colonic tissue integrity, and restoring redox balance. Moreover, APA treatment induced targeted modulation of the gut microbiota, significantly decreasing pro-inflammatory taxa such as *Staphylococcus* and *Enterobacteriaceae*, while partially restoring beneficial genera such as *Roseburia*, which are implicated in anti-inflammatory short-chain fatty acid production. Although overall microbial diversity remained unchanged, these targeted shifts in key taxa indicate a positive modulation of dysbiosis. The short treatment duration likely limited the extent of microbiota recovery, underscoring the need for longer studies to assess sustained effects. Collectively, these findings position APA as a promising multi-target therapeutic agent capable of addressing oxidative stress, inflammation, and microbial imbalance in inflammatory bowel diseases. Future research should focus on elucidating the molecular mechanisms and validating efficacy in clinical settings.

## Figures and Tables

**Table 1:** IC_50_ values for the antioxidant activity of *A. platensis* aqueous extract (APA) and standard references.

	IC_50_ (mg/mL)		
	APA	Trolox	Ascorbic Acid
DPPH• scavenging activity	0.166 ± 0.00	0.081 ± 0.00	0.067 ± 0.00
ABTS•^+^ scavenging activity	0.118 ± 0.00	0.068 ± 0.00	0.072 ± 0.00
Ferrous ion chelating activity	0.939 ± 0.05	0.081 ± 0.00	0.083 ± 0.00

## Data Availability

All data generated or analyzed during this study are included in this published article.

## Supplementary Materials

The following supporting information can be downloaded at https://www.mdpi.com/article/10.3390/cimb47090778/s1.

## Abbreviations

ABTS	2,2′-azino-bis (3-ethylbenzthiazoline-6-sulphonic acid)
APA	*Arthrospira platensis* aqueous extract
CD	Crohn’s Disease
COX-2	Cyclooxygenase-2
DAI	Disease Activity Index
DNBS	Dinitrobenzene Sulfonic Acid
DPPH	1,1-diphenyl-2-picrylhydrazyl
DSS	Dextran Sulfate Sodium
DTNB	5,5′-dithiobis 2-nitrobenzoic
GSH	Reduced Glutathion
HPLC-DAD-ESI-MS	High-Performance Liquid Chromatography coupled with Diode Array Detection and Electrospray Ionization Mass Spectrometry
HTAB	Hexadecyl–Trimethyl–Ammonium Bromide
IBD	Inflammatory Bowel Disease
IC_50_	*Half maximal inhibitory concentration*
IL-6	Interleukin 6
MDA	Malondialdehyde.
MPO	Myeloperoxydase
NF-κB	Nuclear factor kappa B
NO	Nitric Oxide
PGE2	Prostaglandin E2
PMS	Post-Mitochondrial Supernatant.
RNS	Reactive Nitrogen Species
ROS	Reactive Oxygen Species
SCFA	short-chain fatty acid
TBARS	Thiobarbituric acid reactive substances
TNF-α	Tumor Necrosis Factor- Alpha
UC	Ulcerative Colitis

## References

[1] McDowell C., Farooq U., Haseeb M. (2022). Inflammatory Bowel Disease. StatPearls.

[2] Jarmakiewicz-Czaja S., Zielińska M., Sokal A., Filip R. (2022). Genetic and Epigenetic Etiology of Inflammatory Bowel Disease: An Update. Genes.

[3] Bribi N., Yanat B., Ouahmed-Boudaoud H. (2019). Immunopathogenesis of Ulcerative Colitis and Crohn’s Disease. Int. J. Adv. Res. Microbiol. Immunol..

[4] Liu Y., Wang X., Hu C.-A.A. (2017). Therapeutic Potential of Amino Acids in Inflammatory Bowel Disease. Nutrients.

[5] Bribi N., Algieri F., Nogales A., Vezza T., Garrido-Mesa J., Utrilla M., Contreras M.d.M., Maiza F., Segura Carretero A., Rodriguez-Cabezas M. (2016). Intestinal Anti-Inflammatory Effects of Total Alkaloid Extract from *Fumaria capreolata* in the DNBS Model of Mice Colitis and Intestinal Epithelial CMT93 Cells. Phytomedicine.

[6] Cai Z., Wang S., Li J. (2021). Treatment of Inflammatory Bowel Disease: A Comprehensive Review. Front. Med..

[7] Quera R., Núñez P., Sicilia B., Flores L., Gomollón F. (2023). Corticosteroids in Inflammatory Bowel Disease: Are They Still a Therapeutic Option?. Gastroenterol. Hepatol. Engl. Ed..

[8] Elhag D.A., Kumar M., Saadaoui M., Akobeng A.K., Al-Mudahka F., Elawad M., Al Khodor S. (2022). Inflammatory Bowel Disease Treatments and Predictive Biomarkers of Therapeutic Response. Int. J. Mol. Sci..

[9] Mishra R., Dhawan P., Srivastava A.S., Singh A.B. (2020). Inflammatory Bowel Disease: Therapeutic Limitations and Prospective of the Stem Cell Therapy. World J. Stem Cells.

[10] Bribi N., Mohamed Sofiane M., Ouahmed-Boudaoud H. (2023). Intestinal Anti-Inflammatory Effects of *Linum usitatissimum* Alkaloid on Experimental Ulcerative Colitis in BALB/c Mice. Curr. Bioact. Compd..

[11] DeVoss J., Diehl L. (2014). Murine Models of Inflammatory Bowel Disease (IBD): Challenges of Modeling Human Disease. Toxicol. Pathol..

[12] Wen C., Chen D., Zhong R., Peng X. (2024). Animal Models of Inflammatory Bowel Disease: Category and Evaluation Indexes. Gastroenterol. Rep..

[13] Zhou L., Li K., Duan X., Hill D., Barrow C., Dunshea F., Martin G., Suleria H. (2022). Bioactive Compounds in Microalgae and Their Potential Health Benefits. Food Biosci..

[14] Eilam Y., Khattib H., Pintel N., Avni D. (2023). Microalgae—Sustainable Source for Alternative Proteins and Functional Ingredients Promoting Gut and Liver Health. Glob. Chall..

[15] Papadaki S., Tricha N., Panagiotopoulou M., Krokida M. (2024). Innovative Bioactive Products with Medicinal Value from Microalgae and Their Overall Process Optimization through the Implementation of Life Cycle Analysis—An Overview. Mar. Drugs.

[16] Kulshreshtha A., Zacharia A.J., Jarouliya U., Bhadauriya P., Prasad G.B.K.S., Bisen P.S. (2008). Spirulina in Health Care Management. Curr. Pharm. Biotechnol..

[17] Habib A., Hasan M. (2024). A Review on Culture, Production and Use of Spirulina as Food for Humans and Feed for Domestic Animals and Fish. Int. J. Sci. Res. Eng. Manag..

[18] Dillon J.C., Phuc A.P., Dubacq J.P. (1995). Nutritional Value of the Alga Spirulina. World Rev. Nutr. Diet..

[19] Ben Mya O., Souici S., Guenfoud M. (2024). Comparative Analysis of Nutritional and Bioactive Components in Algerian and Egyptian Spirulina from Tamanrasset and Khatatba Regions. Biomass Convers. Biorefinery.

[20] Nuhu A. (2013). Spirulina (*Arthrospira*): An Important Source of Nutritional and Medicinal Compounds. J. Mar. Biol..

[21] Gumbo J., Nesamvuni C. (2017). A Review: Spirulina a Source of Bioactive Compounds and Nutrition. J. Chem. Pharm. Sci..

[22] AlFadhly N.K.Z., Alhelfi N., Altemimi A.B., Verma D.K., Cacciola F., Narayanankutty A. (2022). Trends and Technological Advancements in the Possible Food Applications of Spirulina and Their Health Benefits: A Review. Molecules.

[23] Gentscheva G., Nikolova K., Panayotova V., Peycheva K., Makedonski L., Slavov P., Radusheva P., Petrova P., Yotkovska I. (2023). Application of *Arthrospira platensis* for Medicinal Purposes and the Food Industry: A Review of the Literature. Life.

[24] Wang J., Su L., Zhang L., Zeng J., Chen Q., Deng R., Wang Z., Kuang W., Jin X., Gui S. (2022). *Spirulina platensis* Aqueous Extracts Ameliorate Colonic Mucosal Damage and Modulate Gut Microbiota Disorder in Mice with Ulcerative Colitis by Inhibiting Inflammation and Oxidative Stress. J. Zhejiang Univ. Sci. B.

[25] Tolpeznikaite E., Bartkevics V., Ruzauskas M., Pilkaityte R., Viskelis P., Urbonaviciene D., Zavistanaviciute P., Zokaityte E., Ruibys R., Bartkiene E. (2021). Characterization of Macro-and Microalgae Extracts Bioactive Compounds and Micro-and Macroelements Transition from Algae to Extract. Foods.

[26] Chaves N., Santiago A., Alías J.C. (2020). Quantification of the Antioxidant Activity of Plant Extracts: Analysis of Sensitivity and Hierarchization Based on the Method Used. Antioxidants.

[27] Bibi Sadeer N., Montesano D., Albrizio S., Zengin G., Mahomoodally M.F. (2020). The Versatility of Antioxidant Assays in Food Science and Safety—Chemistry, Applications, Strengths, and Limitations. Antioxidants.

[28] Chai T., Mohan M., Ong H., Wong F. (2014). Antioxidant, Iron-Chelating and Anti-Glucosidase Activities of Typha Domingensis Pers (*Typhaceae*). Trop. J. Pharm. Res..

[29] Martín R., Chain F., Miquel S., Lu J., Gratadoux J.-J., Sokol H., Verdu E.F., Bercik P., Bermúdez-Humarán L.G., Langella P. (2014). The Commensal Bacterium *Faecalibacterium prausnitzii* Is Protective in DNBS-Induced Chronic Moderate and Severe Colitis Models. Inflamm. Bowel Dis..

[30] Merakeb M.S., Bribi N., Ferhat R., Aziez M., Yanat B. (2023). Alkaloids Extract from *Linum usitatissimum* Attenuates 12-OTetradecanoylphorbol-13-Acetate (TPA)-Induced Inflammation and Oxidative Stress in Mouse Skin. Anti-Inflamm. Anti-Allergy Agents Med. Chem..

[31] Aziez M., Suharoschi R., Merakeb M.S., Pop O.L., Ciont C., Ranga F., Ferhat R., Affenai S., Vodnar D.C., Cozma A. (2025). Phenolic Profiling and Bioactive Properties of *Arthrospira platensis* Extract in Alleviating Acute and Sub-Chronic Colitis. Int. J. Mol. Sci..

[32] Rehman I.U., Saleem M., Raza S.A., Bashir S., Muhammad T., Asghar S., Qamar M.U., Shah T.A., Bin Jardan Y.A., Mekonnen A.B. (2024). Anti-Ulcerative Colitis Effects of Chemically Characterized Extracts from Calliandra *Haematocephala* in Acetic Acid-Induced Ulcerative Colitis. Front. Chem..

[33] Aziez M., Bribi N., Mohamed Sofiane M., Riad F., Affenai S. (2024). Intestinal Anti-Inflammatory and Antioxidant Potential of *Arthrospira platensis* Aqueous Extract on DNBS-Induced Colitis in BALB/c Mice. Curr. Chem. Biol..

[34] Avula S.K., Khan A., Halim S.A., Rehman N.U., Karim N., Khan I., Csuk R., Das B., Al-Harrasi A. (2022). Synthesis and Antidepressant-like Effects of New *5-Epi-*Incensole and 5-Epi-Incensole Acetate in Chronic Unpredictable Mild Stress Model of Depression; Behavioural and Biochemical Correlates. Biomed. Pharmacother..

[35] Rathore P., Arora I., Rastogi S., Akhtar M., Singh S., Samim M. (2020). Collagen Nanoparticle-Mediated Brain Silymarin Delivery: An Approach for Treating Cerebral Ischemia and Reperfusion-Induced Brain Injury. Front. Neurosci..

[36] Bradford M.M. (1976). A Rapid and Sensitive Method for the Quantitation of Microgram Quantities of Protein Utilizing the Principle of Protein-Dye Binding. Anal. Biochem..

[37] Rodríguez-Díaz C., Martín-Reyes F., Taminiau B., Ho-Plágaro A., Camargo R., Fernandez-Garcia F., Pinazo-Bandera J., Toro-Ortiz J.P., Gonzalo M., López-Gómez C. (2023). The Metagenomic Composition and Effects of Fecal-Microbe-Derived Extracellular Vesicles on Intestinal Permeability Depend on the Patient’s Disease. Int. J. Mol. Sci..

[38] Rather I.A., Bajpai V.K., Ching L.L., Majumder R., Nam G.-J., Indugu N., Singh P., Kumar S., Hajrah N.H., Sabir J.S.M. (2020). Effect of a Bioactive Product SEL001 from *Lactobacillus sakei* Probio65 on Gut Microbiota and Its Anti-Colitis Effects in a TNBS-Induced Colitis Mouse Model. Saudi J. Biol. Sci..

[39] Jensen G.S., Attridge V.L., Beaman J.L., Guthrie J., Ehmann A., Benson K.F. (2015). Antioxidant and Anti-Inflammatory Properties of an Aqueous Cyanophyta Extract Derived from *Arthrospira platensis*: Contribution to Bioactivities by the Non-Phycocyanin Aqueous Fraction. J. Med. Food.

[40] Grover P., Bhatnagar A., Kumari N., Narayan Bhatt A., Kumar Nishad D., Purkayastha J. (2021). C-Phycocyanin-a Novel Protein from *Spirulina platensis*—In Vivo Toxicity, Antioxidant and Immunomodulatory Studies. Saudi J. Biol. Sci..

[41] Shalaby E., Shanab S. (2013). Comparison of DPPH and ABTS Assays for Determining Antioxidant Potential of Water and Methanol Extracts of *Spirulina platensis*. Indian J. Mar. Sci..

[42] Bellahcen T.O., AAmiri A., Touam I., Hmimid F., Amrani A.E., Cherif A., Cherki M. (2020). Evaluation of Moroccan Microalgae: *Spirulina platensis* as a Potential Source of Natural Antioxidants. J. Complement. Integr. Med..

[43] Coskun Z.K., Kerem M., Gurbuz N., Omeroglu S., Pasaoglu H., Demirtas C., Lortlar N., Salman B., Pasaoglu O.T., Turgut H.B. (2011). The Study of Biochemical and Histopathological Effects of Spirulina in Rats with TNBS-Induced Colitis. Bratisl. Lek. Listy.

[44] Morsy M.A., Gupta S., Nair A.B., Venugopala K.N., Greish K., El-Daly M. (2019). Protective Effect of *Spirulina platensis* Extract against Dextran-Sulfate-Sodium-Induced Ulcerative Colitis in Rats. Nutrients.

[45] Guo W., Zhu S., Feng G., Wu L., Feng Y., Guo T., Yang Y., Wu H., Zeng M. (2020). Microalgae Aqueous Extracts Exert Intestinal Protective Effects in Caco-2 Cells and Dextran Sodium Sulphate-Induced Mouse Colitis. Food Funct..

[46] Garcia F.A.d.O., Sales-Campos H., Yuen V.G., Machado J.R., Viana G.S.d.B., Oliveira C.J.F., McNeill J.H. (2020). *Arthrospira* (Spirulina) Platensis Attenuates Dextran Sulfate Sodium-Induced Colitis in Mice by Suppressing Key Pro-Inflammatory Cytokines. Korean J. Gastroenterol..

[47] Ilieva Y., Zaharieva M.M., Najdenski H., Kroumov A.D. (2024). Antimicrobial Activity of *Arthrospira* (Former Spirulina) and Dunaliella Related to Recognized Antimicrobial Bioactive Compounds. Int. J. Mol. Sci..

[48] Masoudi-Sobhanzadeh Y., Pourseif M.M., Khalili-Sani A., Jafari B., Salemi A., Omidi Y. (2023). Deciphering Anti-Biofilm Property of *Arthrospira platensis*-Origin Peptides against Staphylococcus aureus. Comput. Biol. Med..

[49] Caparrós E., Wiest R., Scharl M., Rogler G., Gutiérrez Casbas A., Yilmaz B., Wawrzyniak M., Francés R. (2021). Dysbiotic Microbiota Interactions in Crohn’s Disease. Gut Microbes.

[50] Crothers J.W., Chu N.D., Nguyen L.T.T., Phillips M., Collins C., Fortner K., Del Rio-Guerra R., Lavoie B., Callas P., Velez M. (2021). Daily, Oral FMT for Long-Term Maintenance Therapy in Ulcerative Colitis: Results of a Single-Center, Prospective, Randomized Pilot Study. BMC Gastroenterol..

[51] Chen J., Vitetta L. (2020). Butyrate in Inflammatory Bowel Disease Therapy. Gastroenterology.

[52] Recharla N., Geesala R., Shi X.-Z. (2023). Gut Microbial Metabolite Butyrate and Its Therapeutic Role in Inflammatory Bowel Disease: A Literature Review. Nutrients.

