# Supplementary material for: Pharmacological Potential of Arthrospira platensis in Mitigating Sub-Chronic Colitis: Redox Homeostasis and Gut Microbiota Modulation
Source: Curr Issues Mol Biol. 2025 Sep 19;47(9):778. doi: 10.3390/cimb47090778 (PMC12468418; doi:10.3390/cimb47090778)
Supplement: Supplementary file 1 [file cimb-47-00778-s001.zip › cimb-3820398-20251021-supplementary.pdf]

Pharmacological Potential of *Arthrospira platensis* in Mitigating Sub-chronic Colitis: Redox Homeostasis and Gut Microbiota Modulation

Meriem Aziez [1,†], Betitera Yanat [1,†], Cristina Rodriguez-Diaz [2,3], Ramona Suharoschi [4,*], Romana Vulturar [5], Simona-Codruta Heghes [6], Nawel Guenaoui [7], Awadh M. Ali [8], Eduardo Garcia-Fuentes [2,3,9] and Noureddine Bribi [1,*]

1 Laboratory of Plant Biotechnology and Ethnobotany, Faculty of Nature and Life Sciences, University of Bejaia, Bejaia 06000, Algeria; meriem.aziez@univ-bejaia.dz (M.A.); betitera.yanat@u-pec.fr (B.Y.)
2 Instituto de Investigación Biomédica de Málaga y Plataforma en Nanomedicina-IBIMA Plataforma BIONAND, 29010 Málaga, Spain; cris.rdrz@gmail.com (C.R.-D.); edugf1@gmail.com (E.G.-F.)
3 Servicio de Aparato Digestivo, Hospital Universitario Virgen de la Victoria, 29010 Málaga, Spain
4 Molecular Nutrition and Proteomics Lab, Bld. Life Science Institute, Department of Food Science, University of Agricultural Science and Veterinary Medicine, 3-5 Calea Mănăstur, 400372 Cluj-Napoca, Romania
5 Department of Cell and Molecular Biology, "Iuliu Hațieganu" University of Medicine and Pharmacy, 6 Louis Pasteur St., 400349 Cluj-Napoca, Romania; romanavulturar@gmail.com
6 Department of Drug Analysis, "Iuliu Hațieganu" University of Medicine and Pharmacy, 6 Louis Pasteur Street, 400349 Cluj-Napoca, Romania; cmaier@umfcluj.ro
7 Applied Biochemistry Laboratory, Faculty of Natural and Life Sciences, University of Bejaia, Bejaia 06000, Algeria; nawel.guenaoui@univ-bejaia.dz
8 Department of Pharmaceutical Chemistry, College of Pharmacy, King Saud University, P.O. Box. 2457, Riyadh 11451, Saudi Arabia; aali1@ksu.edu.sa
9 Centro de Investigación Biomédica en Rehd de Enfermedades Hepáticas y Digestivas (CIBERehd), 29010 Málaga, Spain
* Correspondence: ramona.suharoschi@usamvcluj.ro (R.S.); noureddine.bribi@univ-bejaia.dz (N.B.)
† These authors contributed equally to this work.

Table S1. HPLC-DAD-ESI-MS Identification of Phenolic Compounds in APA Extract.

Phenolic Compound		R_t (min)	UV λ_{max} (nm)	$[M+H]^+$ (m/z)	Content (mg. g^{-1}APA)
1	Pirogallol	2.81	270	127	2,57
2	3-Hydroxybenzoic acid	3.32	270	139	1,096
3	2,4 Dihydroxybenzoic acid	3.57	270	155	1,646
4	3,5 Dihydroxybenzoic acid	4.32	270	155	1,946
5	Gallic acid	5.10	270	171	1,158
6	Protocatechuic acid	9.29	280	155	0,098
7	Chlorogenic acid	12.20	330	355	0,159
8	Vanilic acid	13.04	280	169	0,009
9	p-Coumaric acid	16.82	331	165	0,07
10	Ferulic acid	17.68	331	195	0,107
	**Total phenolics**				**8,859**

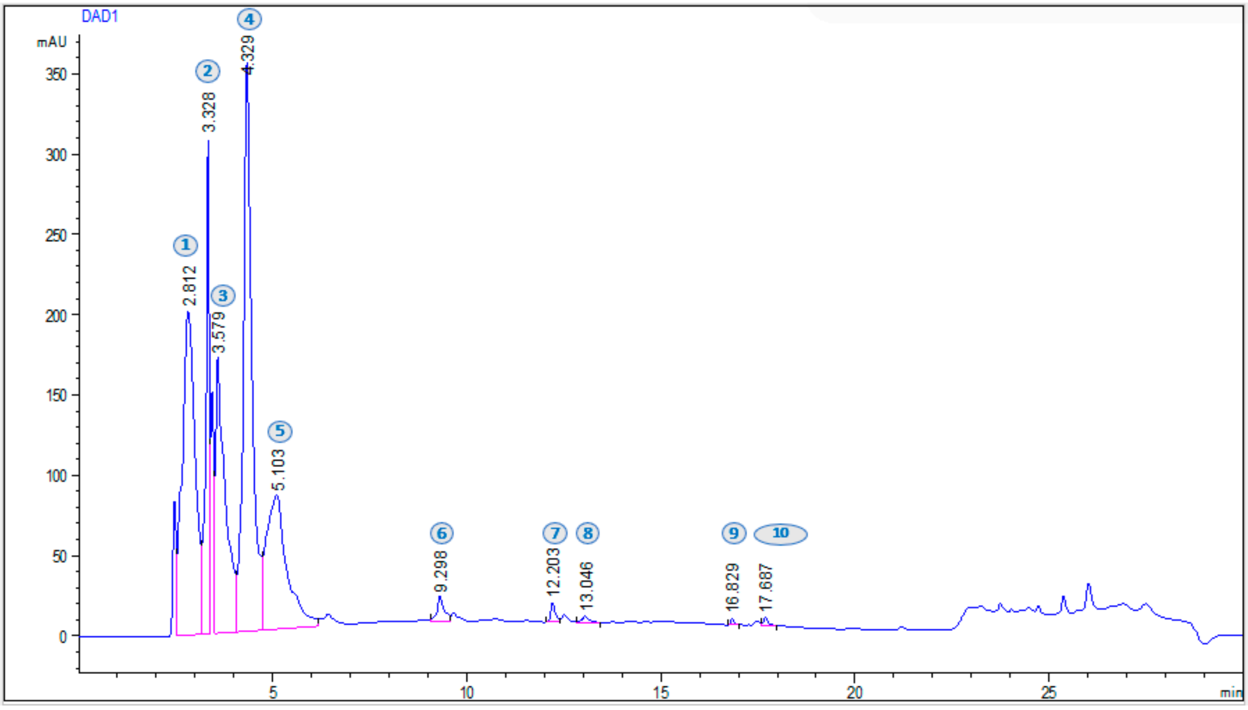

Figure S1. Representative DAD chromatogram of phenolic compounds presents in the aqueous extract of *Arthrospira platensis* (APA) obtained by HPLC-DAD-ESI-MS.
